# SFC-YOLO: An Accuracy-Enhanced and Parameter-Efficient YOLO Framework for Small Vehicle Detection in Aerial Images

**DOI:** 10.3390/s26154923

**Published:** 2026-08-04

**Authors:** Jinwei Zuo, Qiyi He, Fulin Liu, Jieying Liu, Minlong Hu, Yiguo Wang, Tingting Zhang, Yiming Yang, Qiao Lin

**Affiliations:** 1 School of Computer Science, Hubei University of Technology, Wuhan 430068, China; 2 Hubei Luojia Laboratory, Wuhan 430079, China; 3 School of Computer Science, University of Nottingham Ningbo China, Ningbo 315100, China

**Keywords:** aerial image detection, small vehicle detection, YOLO11n, dynamic upsampling, hidden-state attention, parameter-efficient detection, intelligent transportation

## Abstract

Small vehicle detection in aerial images is important for intelligent transportation, low-altitude inspection, urban monitoring, and vision-based sensing systems. Vehicles in aerial images often occupy few pixels and are affected by complex backgrounds, shadows, viewpoint changes, weak texture, and similar class appearances, which can cause missed detections and false alarms. To address these issues, this paper proposes SFC-YOLO, an accuracy-enhanced and parameter-efficient framework based on YOLO11n. A Feature Complementary Block (FCB) is placed at the P5/32 high-level feature stage to enhance local-detail and semantic-context compensation; a Dynamic Feature Alignment Upsampling unit (DFAU) is inserted into the first P5-to-P4 upsampling path to improve content-adaptive feature alignment; and a P5-only Hidden-State Attention (HSA) module is used in the final P5 detection branch to strengthen global semantic interaction. Experiments on the VEDAI eight-class vehicle dataset show that SFC-YOLO reduces the parameter count from 2.584 M to 2.466 M while improving the five-run mean Precision from 0.602 to 0.665, Recall from 0.599 to 0.608, and mAP50 from 0.614 to 0.652. Since the GFLOPs increase from 6.3 to 8.4, SFC-YOLO should be interpreted as a parameter-reduced but not FLOP-reduced framework. The main trade-off is improved detection accuracy with fewer trainable parameters at the cost of higher theoretical computation. Five repeated experiments yield an average mAP50 of 0.6523 ± 0.0117, quantifying the run-to-run variation under the current training protocol.

## 1. Introduction

Vehicle detection in aerial imagery aims to automatically locate and recognize vehicles from images collected by aircraft, unmanned aerial vehicles, low-altitude inspection platforms, and other visual sensing systems. It provides a visual foundation for intelligent transportation, road-condition perception, parking-resource monitoring, emergency response, and fine-grained urban management. With the rapid development of low-altitude remote-sensing platforms and high-resolution visual sensors, large-area and multi-view road images have become increasingly available for vehicle distribution monitoring and traffic-state analysis. Remote-sensing object detection has been widely studied because objects in aerial images often exhibit large variations in scale, orientation, appearance, and background complexity [1,2,3,4].

Compared with object detection in natural scenes, aerial-image vehicle detection has distinct characteristics. First, vehicles usually occupy a small proportion of the image, and repeated downsampling in deep neural networks can weaken boundaries, corners, and local texture cues. Second, aerial backgrounds are complex, and vehicles may have responses similar to roofs, road markings, shadows, small facilities, and ground textures. Third, in parking lots, congested roads, and industrial areas, vehicles are often densely distributed and have diverse orientations. Detectors therefore need accurate localization, robust cross-scale feature fusion, and stable semantic discrimination [5,6,7].

Deep learning has substantially promoted remote-sensing object detection. Two-stage detectors often provide strong localization ability but have relatively high computational cost, while single-stage and transformer-based detectors provide alternative accuracy–efficiency trade-offs [8,9,10,11,12]. Single-stage detectors represented by the YOLO series achieve a favorable balance between speed and accuracy and are widely used in real-time perception and edge-deployment scenarios. YOLO11n has a relatively low parameter count and a mature training and deployment ecosystem, making it suitable as a baseline framework for aerial sensing applications with controlled model size. However, directly applying YOLO11n to aerial vehicle detection still suffers from insufficient high-level small-object representation, spatial misalignment during multiscale upsampling, and inadequate high-level semantic context modeling under complex backgrounds.

To address these issues, this paper proposes SFC-YOLO, an accuracy-enhanced and parameter-efficient YOLO framework for small vehicle detection in aerial images. Rather than stacking modules across the entire network, SFC-YOLO introduces three targeted structural modifications according to the P3/P4/P5 detection hierarchy of YOLO11n. FCB enhances complementary high-level features at P5/32, DFAU improves dynamic P5-to-P4 cross-scale alignment, and HSA introduces hidden-state semantic interaction in the final P5 detection branch.

The main contributions are summarized as follows:

1. We propose SFC-YOLO, a bottleneck-oriented enhancement of YOLO11n for small vehicle detection in aerial images. By introducing FCB, DFAU, and P5-only HSA at three task-critical positions, the framework targets high-level detail attenuation, P5-to-P4 spatial misalignment, and limited semantic interaction under complex aerial backgrounds.
2. We provide a controlled validation of SFC-YOLO on VEDAI and DOTA-v1.0 HBB. On VEDAI, SFC-YOLO improves mAP50 from 0.614 to 0.652 while reducing the parameter count from 2.584 M to 2.466 M; on DOTA-v1.0 HBB 1024-patch validation, it improves mAP50 from 0.69505 to 0.70033. It should be emphasized that SFC-YOLO is parameter-reduced rather than FLOP-reduced: the parameter count decreases by 4.55%, whereas GFLOPs increase by 33.33%. Therefore, the intended trade-off is accuracy versus parameter count, not accuracy versus theoretical computation. Repeated experiments, ablation analysis, and complexity evaluation are further used to examine run-to-run variation, module contribution, and computational trade-offs.

## 2. Related Work

### 2.1. Remote Sensing Vehicle Detection

Remote sensing vehicle detection commonly faces small objects, dense distributions, scale variations, and complex backgrounds. Traditional approaches relied on hand-crafted features and classifiers, and their generalization ability was limited by scene variation. With the development of convolutional neural networks, aerial vehicle detection has gradually shifted to end-to-end object detection frameworks, where multilayer feature extraction and multiscale prediction improve detection performance under complex scenes [1,2,7].

Public aerial and drone-view benchmarks, including VEDAI, DOTA, VisDrone, xView, and FAIR1M, have promoted the development and evaluation of remote-sensing object detectors [5,6,13,14,15,16,17]. Existing studies usually improve detectors from the perspectives of small-object feature enhancement, class imbalance handling, oriented bounding-box modeling, multiscale feature fusion, super-resolution assistance, slicing-based inference, and resource-constrained deployment [18,19,20,21,22,23]. This work focuses on horizontal-bounding-box vehicle detection and aims to improve feature representation and feature fusion for YOLO11n in aerial-image small vehicle detection.

### 2.2. YOLO-Based Efficient Detection

The YOLO series adopts a single-stage detection paradigm and achieves a favorable balance between detection accuracy and inference speed [24,25,26,27,28,29,30,31]. Small-parameter YOLO models reduce model size by decreasing network width, reducing depth, or using efficient convolutional structures, which makes them suitable when parameter scale must be controlled. Nevertheless, small-parameter models often have insufficient feature capacity in aerial small-object scenes, resulting in missed detections.

This paper uses YOLO11n as the baseline and performs targeted enhancement of high-level feature representation, cross-scale fusion, and semantic context modeling without significantly expanding the parameter scale. Different from simply increasing model capacity, SFC-YOLO emphasizes the correspondence between module placement and task bottlenecks.

Recent YOLO11n-based studies have also explored accuracy–parameter trade-offs for UAV small-object detection. For example, Zhu and Xie proposed an enhanced YOLOv11n model for UAV imagery by introducing a Multiscale Edge-Feature Adaptive Selection (MSEAF) module, ScalCat/Scal3DC-based neck reconstruction, a P2 small-object detection head, and a shared reparameterized lightweight detection head [32]. Their work further indicates that improving YOLO11n for UAV small-object detection requires balancing shallow detail preservation, multiscale feature fusion, and parameter control. Different from that study, SFC-YOLO focuses on aerial small vehicle detection and investigates P5 high-level feature compensation, P5-to-P4 dynamic feature alignment, and P5-only hidden-state semantic interaction under a controlled YOLO11n-based framework.

### 2.3. Feature Fusion, Dynamic Upsampling, and Attention

Multiscale feature fusion is critical for object detection. Conventional feature pyramids and path-aggregation structures combine deep semantic information with shallow spatial details through upsampling, concatenation, or weighted fusion [33,34,35,36,37,38]. However, fixed interpolation changes only the feature-map size and cannot adapt sampling locations according to image content, which may cause misalignment between high-level semantic responses and mid-level spatial details. Dynamic upsampling and learnable spatial sampling methods learn sampling offsets or sampling weights to improve spatial reconstruction, which is suitable for small-object localization [39,40,41,42,43].

Attention and state-space modeling methods can enhance channel, spatial, long-range dependency, and global-context representation in visual models [44,45,46,47,48,49,50,51]. However, directly introducing complex attention into high-resolution shallow features can substantially increase computational cost. In this work, HSA is placed only in the P5 high-level branch and performs self-attention in a compact hidden-state space, thereby controlling additional computation while improving semantic discrimination.

## 3. Materials and Methods

### 3.1. Task Definition and Design Objective

This study focuses on small vehicle detection in aerial images. In this task, vehicles usually have small pixel occupancy, weak visual texture, large orientation variation, and strong background interference. After multiple downsampling stages, the boundaries, corners, and local texture responses of small vehicles are easily weakened. Meanwhile, road markings, building edges, shadows, and ground facilities may generate local structures similar to vehicles. Therefore, the detector should preserve spatial localization capability while improving high-level semantic representation for small objects and their contextual relationships.

YOLO11n is adopted as the baseline framework. The design objective is to improve detection accuracy and parameter efficiency for aerial-image small vehicle detection. Since dynamic sampling and hidden-state interaction introduce additional computation, SFC-YOLO is not positioned as a low-FLOPs detector. Instead, it is defined as a parameter-efficient accuracy-enhanced framework under a controlled parameter scale. Parameter count, GFLOPs, and practical inference speed are reported jointly in the experimental section.

SFC-YOLO follows a targeted structural-enhancement strategy rather than a full-network stacking strategy. The three modifications act on high-level feature compensation, P5-to-P4 alignment upsampling, and final P5 contextual interaction, respectively, to alleviate insufficient deep small-object representation, cross-scale fusion misalignment, and limited high-level semantic discrimination under complex backgrounds.

### 3.2. Baseline Bottlenecks in Aerial-Image Small Vehicle Detection

YOLO11n uses a three-scale detection structure, where P3, P4, and P5 correspond to different downsampling scales. P3 retains higher spatial resolution and is suitable for small-object localization; P4 balances spatial details and semantic representation; and P5 has stronger semantic abstraction but lower spatial resolution. For aerial vehicle detection, the main bottlenecks are concentrated in three aspects.

First, small-object representation in deep features is insufficient. Vehicles occupy few pixels in aerial images, and deep downsampling weakens boundaries and local textures, making high-level semantic features less capable of preserving small-object details. Second, spatial alignment errors exist when high-level semantic features are fused into mid-level features. Fixed upsampling does not adjust sampling locations according to image content, and even small spatial offsets may influence classification confidence and bounding-box localization. Third, the high-level detection branch has limited ability to model context under complex backgrounds, and local convolutional responses can be disturbed by road textures, building edges, parking lines, and shadows.

Based on these bottlenecks, SFC-YOLO enhances the P5 high-level feature stage, the P5-to-P4 fusion path, and the final P5 detection branch. This strategy allows each module to correspond to a specific error source and concentrates additional computation on low-resolution or key fusion paths.

### 3.3. Overall Framework of SFC-YOLO

**Figure 1:**
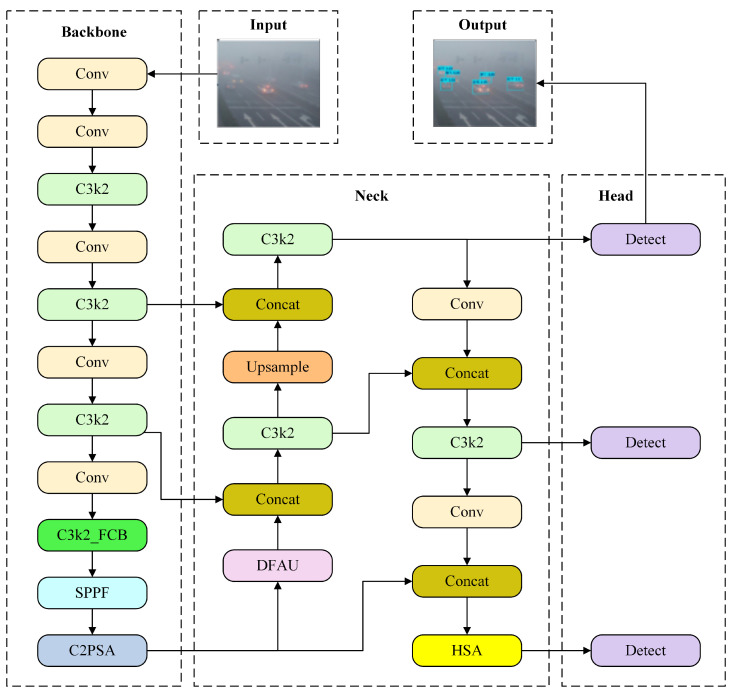
Overall framework of SFC-YOLO for aerial-image small vehicle detection.

SFC-YOLO retains the three-scale detection framework of YOLO11n and introduces three functionally complementary structural units at key positions. The overall framework is shown in Figure 1. FCB enhances local details and semantic context in P5/32 high-level features. DFAU improves content-adaptive spatial sampling in the P5-to-P4 fusion path. HSA strengthens high-level semantic interaction in the final P5 detection branch.

(1) $$P_{i}=F_{i}\left(I\right),\;i\in \left\{3,4,5\right\},$$

where *I* denotes the input image, and $P_{3}$, $P_{4}$, and $P_{5}$ denote feature layers with different downsampling scales. SFC-YOLO does not change the basic prediction form of the three-scale detection head. Instead, it improves the generation and fusion of key features to provide higher-quality representations to the detection head. The positions, functions, and addressed bottlenecks of the three modifications are summarized in Table 1.

This design follows three principles. First, the modifications target real error sources in aerial-image small-object detection rather than simply increasing the number of modules. Second, the parameter scale remains controlled to avoid exchanging accuracy improvement for a substantially larger network capacity. Third, complex operations are preferentially placed on low-resolution features or key fusion paths to reduce their impact on overall computation and training stability.

### 3.4. Feature Complementary Block for High-Level Feature Compensation

FCB is designed to alleviate the attenuation of local small-object details in P5/32 high-level features. Vehicle targets in aerial images require edges, corners, and short-range texture cues for localization, while semantic context is also needed to suppress background interference such as roads, roofs, and shadows. A single convolutional path is insufficient to simultaneously maintain local details and semantic abstraction. Therefore, FCB adopts complementary modeling with a local-detail branch and a semantic-context branch.

FCB is implemented through a C3k2-style wrapper at the P5/32 stage. In the reported YOLO11n-scale configuration, the wrapper receives and outputs 256-channel feature maps. Its internal C3k path contains two FCM cores, each operating on a 64-channel hidden representation.

Let $X_{h}\in R^{B\times C_{h}\times H\times W}$ denote the hidden feature entering an FCM core. It is divided into a local-detail branch $X_{L}$ and a semantic-context branch $X_{S}$ using a fixed 1:3 channel ratio:

(2) $$X_{h}=\mathrm{Concat}\left(X_{L},X_{S}\right),\;X_{L}\in R^{B\times C_{h}/4\times H\times W},\;X_{S}\in R^{B\times 3C_{h}/4\times H\times W}.$$

In the reported P5 implementation, $C_{h}=64$; therefore, the local-detail and semantic-context branches contain 16 and 48 channels, respectively.

The local-detail branch applies two consecutive 3×3 convolutions with stride 1 and padding 1, followed by a 1×1 projection to restore the channel dimension. The semantic-context branch uses a 1×1 projection. These branch transformations are implemented with convolution, batch normalization, and SiLU activation. The transformed branch features and their complementary weights are expressed as

(3) $$\begin{array}{cc} F_{L} & ={\mathrm{Conv}}_{1\times 1}\left({\mathrm{Conv}}_{3\times 3}\left({\mathrm{Conv}}_{3\times 3}\left(X_{L}\right)\right)\right),\\ F_{S} & ={\mathrm{Conv}}_{1\times 1}\left(X_{S}\right),\\ A_{S} & =\sigma \left(\mathrm{BN}\left({\mathrm{Conv}}_{1\times 1}\left(F_{S}\right)\right)\right),\\ A_{C} & =\sigma \left(\mathrm{GAP}\left({\mathrm{DWConv}}_{3\times 3}\left(F_{L}\right)\right)\right), \end{array}$$

where $A_{S}$ is a spatial weight generated from the semantic-context branch and $A_{C}$ is a channel weight generated from the local-detail branch. The depthwise convolution uses a 3×3 kernel with stride 1, padding 1, and one group per channel. The complementary branch responses are fused as

(4) $$Y_{\mathrm{FCB}}={\mathrm{Conv}}_{1\times 1}\left(A_{S}⊙F_{L}+A_{C}⊙F_{S}\right),$$

where ⊙ denotes element-wise multiplication with spatial or channel broadcasting. The final 1×1 fusion projection is followed by batch normalization and SiLU activation.

Unlike SE, CBAM, and Coordinate Attention, which recalibrate a single feature tensor through channel and/or spatial attention [44,45,50], FCB first separates local-detail and semantic-context representations and then performs bidirectional cross-branch modulation. The semantic-context branch spatially guides the local-detail response, whereas the local-detail branch constrains channel selection in the semantic-context representation. Thus, the novelty of FCB lies in its asymmetric branch allocation and cross-branch complementary weighting rather than in applying another sequential attention operation to an unchanged feature tensor.

**Figure 2:**
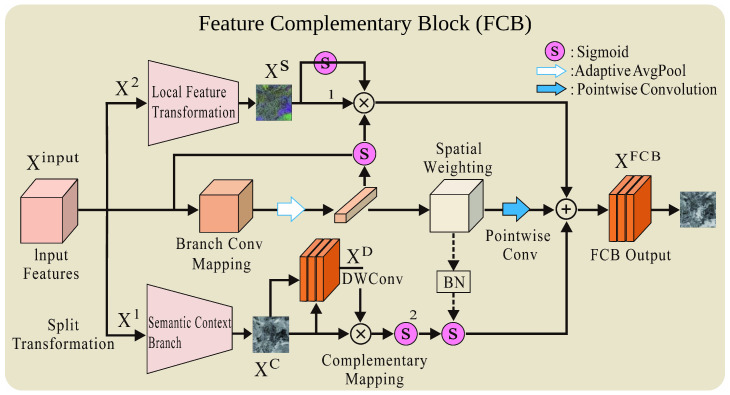
Structure of the Feature Complementary Block used for high-level feature compensation. Magenta circles labeled S denote Sigmoid operations; open light-blue arrows denote adaptive average pooling; filled blue arrows denote pointwise convolution; black solid arrows indicate the main tensor flow; black dashed arrows indicate the normalization path; and + and × denote element-wise addition and multiplication, respectively.

FCB is placed only at the P5/32 high-level feature stage because this scale provides stronger category semantics and lower spatial resolution. This placement concentrates the additional feature modulation on the deep representation without repeatedly introducing the module into high-resolution shallow stages. The structure of FCB is illustrated in Figure 2.

### 3.5. Dynamic Feature Alignment Upsampling for P5-to-P4 Fusion

DFAU addresses spatial alignment in the P5-to-P4 fusion path. P5 features contain stronger semantic information but have lower spatial resolution, whereas P4 features retain more mid-level spatial structure. Conventional nearest-neighbor interpolation changes the feature-map size using fixed sampling positions and therefore cannot adapt the reconstruction process to image content. For small vehicles, even a limited spatial offset may cause inconsistency between high-level semantic responses and target boundaries.

DFAU is implemented using a point-sampling-based dynamic upsampling formulation derived from DySample [43]. Instead of predicting a spatially varying convolution kernel, it predicts residual sampling offsets through a 1×1 convolution. For the input feature *X*, the offset field and dynamic sampling grid are constructed as

(5) $$\Delta P=0.25\;{\mathrm{Conv}}_{1\times 1}\left(X\right)+P_{\mathrm{init}},\;G_{\mathrm{dyn}}=\mathrm{PixelShuffle}\left(\mathrm{Normalize}(G_{\mathrm{base}}+\Delta P)\right),$$

where $P_{\mathrm{init}}$ denotes the regular initial offset pattern, $G_{\mathrm{base}}$ denotes the base coordinate grid, and the factor 0.25 constrains the offset magnitude during early optimization. The aligned output is obtained by grouped differentiable bilinear sampling:

(6) $$Y_{\mathrm{DFAU}}={\mathrm{GridSample}}_{\mathrm{bilinear}}\left(X_{\mathrm{group}},G_{\mathrm{dyn}}\right).$$

In the reported implementation, DFAU uses an upsampling scale of 2, the low-resolution-to-high-resolution coordinate-generation style (style=lp), four sampling groups, and no additional dynamic-scope branch (dyscope=False). The offset-prediction layer is a 1×1 convolution initialized from a zero-mean normal distribution with a standard deviation of 0.001, so that the initial operation remains close to regular upsampling. Coordinate normalization follows the convention of differentiable grid sampling, and bilinear sampling is performed with padding_mode=border and align_corners=False. DFAU preserves the feature-channel dimension and changes only the spatial resolution from $H_{5}\times W_{5}$ to $2H_{5}\times 2W_{5}$.

**Figure 3:**
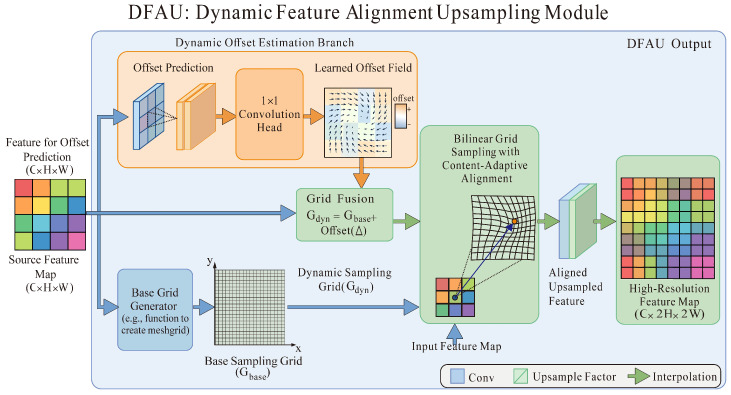
Dynamic Feature Alignment Upsampling module for content-adaptive P5-to-P4 feature alignment. Blue arrows denote source-feature and base-grid flow, orange arrows denote the learned-offset branch, green arrows denote interpolation and aligned-feature flow, and the colored points indicate regular and dynamically shifted sampling locations.

Only the first P5-to-P4 upsampling path uses DFAU, while the subsequent P4-to-P3 path retains nearest-neighbor upsampling. This targeted placement improves the transmission of deep semantic responses to the P4 spatial scale without applying dynamic sampling repeatedly to higher-resolution feature maps. The workflow of DFAU is shown in Figure 3.

### 3.6. P5-Only Hidden-State Attention for Global Semantic Interaction

HSA is used to enhance high-level semantic interaction in the final P5 detection branch. In aerial images, vehicles are often close to road textures, building edges, parking lines, shadows, and small ground facilities. These background structures can resemble vehicle targets in local morphology. Local convolutional operations can extract edges, textures, and short-range spatial patterns, but contextual modeling mainly depends on stacked receptive fields and local response propagation. When object scale is small and background structure is complex, relying only on local responses may cause false alarms or unstable category discrimination. Therefore, the final high-level detection branch requires stronger cross-region semantic interaction.

The P5-only placement is deliberate. Directly introducing global attention into P3 or P4 high-resolution feature maps would substantially increase computation and could weaken computational control. In contrast, P5 has lower spatial resolution and stronger semantic abstraction, making it suitable for contextual interaction at a controllable cost. Therefore, HSA is restricted to the final P5 detection branch to enhance high-level semantics without modifying the basic shallow and mid-level fusion structure.

The hidden-state construction is motivated by hidden-state mixer and state-space duality formulations, in which the spatial sequence is first compressed into a compact latent-state representation before global interaction [51]. Unlike standard spatial self-attention [47], which directly computes pairwise interactions among all $L=H_{5}W_{5}$ spatial tokens, HSA performs self-attention over a fixed set of N=64 compact hidden states. Therefore, the attention matrix is constructed in the hidden-state domain rather than over the complete spatial sequence.

In the reported implementation, the state-variable projection maps $d_{\mathrm{model}}$ channels to 3N channels, which are subsequently divided into $B_{s}$, $C_{s}$, and Δt. The hidden/gating projection maps $d_{\mathrm{model}}$ channels to $2d_{\mathrm{inner}}$, where $d_{\mathrm{inner}}=d_{\mathrm{model}}$ because the state-space expansion ratio is 1. The QKV projection maps $d_{\mathrm{inner}}$ to $3d_{\mathrm{inner}}$, and the attention-output projection preserves $d_{\mathrm{inner}}$. Four attention heads are used, and both attention and projection dropout rates are set to zero.

The following formulation summarizes the HSM-SSD-HSA mixer core inside the P5-only HSA block, while the surrounding C3k/C2f wrapper remains unchanged. Let the input P5 feature be

(7) $$P_{5}\in R^{B\times C\times H_{5}\times W_{5}}.$$

It is first flattened and normalized as

(8) $$X=\mathrm{LN}\;\left(\mathrm{Flatten}(P_{5})\right),\;X\in R^{B\times d_{\mathrm{model}}\times L},\;L=H_{5}W_{5}.$$

The state-space variables and the compact hidden-state representation are constructed by

(9) $$\begin{array}{cc} \left[B_{s},C_{s},\Delta t\right] & =\mathrm{Split}\;\left({\mathrm{DWConv}}_{3\times 3}\left({\mathrm{Proj}}_{1\times 1}\left(X\right)\right)\right),\\ A & ={\mathrm{Softmax}}_{L}\left(\Delta t+A_{0}\right),\\ H_{0} & =X{\left(A⊙B_{s}\right)}^{T}, \end{array}$$

where $B_{s},C_{s},\Delta t,A\in R^{B\times N\times L}$, $H_{0}\in R^{B\times d_{\mathrm{model}}\times N}$, and N=64.

The hidden representation is projected into a hidden-state branch *H* and a gating branch *G*. Self-attention is then performed over the compact hidden states:

(10) $$\begin{array}{cc} \left[H,G\right] & =\mathrm{Split}\;\left({\mathrm{Proj}}_{h/g}\left(H_{0}\right)\right),\\ \left[Q,K,V\right] & ={\mathrm{Proj}}_{qkv}\left(H\right),\\ \mathrm{Attn}(H)\; & =W_{p}\left[\mathrm{Softmax}\left(\frac{QK^{T}}{\sqrt{d_{h}}}\right)V\right]. \end{array}$$

Finally, the gated hidden state, the learnably weighted attention response, and the state-residual branch are fused and reconstructed as

(11) $$\begin{array}{cc} \tilde{H}\; & =H⊙\mathrm{SiLU}(G)+\lambda \;\mathrm{Attn}(H)+D\;H,\\ Y_{\mathrm{HSA}}\; & =\mathrm{Reshape}\;\left(W_{o}\left(\tilde{H}\right)C_{s}\right). \end{array}$$

**Figure 4:**
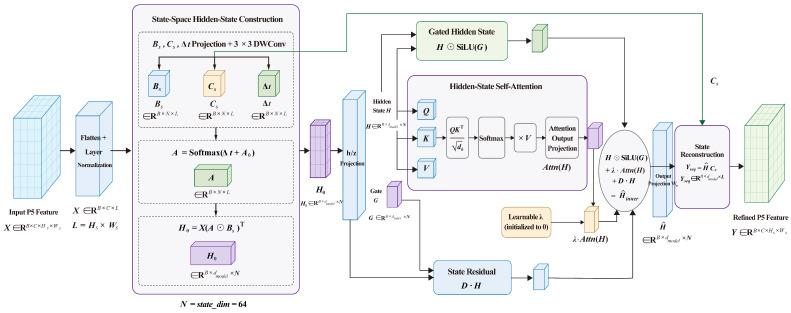
Detailed implementation-level workflow of the P5-only Hidden-State Attention (HSA) mixer. The input P5 feature is flattened and normalized before the state variables $B_{s}$, $C_{s}$, and Δt are generated through projection and depthwise convolution. The compact hidden states are processed by gated hidden-state modeling, hidden-state self-attention, and a state-residual branch. Their responses are fused through a learnable attention coefficient λ, followed by output projection, state reconstruction, and reshaping to obtain the refined P5 feature. Blue blocks denote hidden-state features and projections, purple blocks denote compact hidden states, gating, and attention processing, green blocks and arrows denote state variables, gated features, and state reconstruction, orange blocks denote the learnable attention-weight branch, and black arrows indicate tensor flow.

Here, λ is initialized to zero. In the reported configuration, N=64, the number of attention heads is 4, the state-space expansion ratio is 1, and both attention and projection dropout rates are set to 0. By performing self-attention over compact hidden states rather than the complete spatial feature map, HSA introduces long-range semantic interaction at a controllable computational cost. The detailed workflow is illustrated in Figure 4.

### 3.7. Network Architecture and Implementation Details of the Proposed Modules

To improve clarity and reproducibility, this subsection summarizes the network-level modification positions and the implementation details of the proposed FCB, DFAU, and HSA modules. The three modules are inserted only at the targeted positions described in the architecture, and no additional detection head or prediction scale is introduced. The output feature dimensions of the modified paths are kept compatible with the original YOLO11n neck and detection head. Table 2 summarizes the architecture-level modifications and parameter-count accounting, while Table 3 provides the module-level implementation details.

The detailed configurations and forward implementations of FCB, DFAU, and HSA are further summarized in Table 3.

As pseudocode-level guidance, the forward procedures of the three modules can be summarized as follows. For FCB, each internal FCM core divides its 64-channel hidden feature into a 16-channel local-detail branch and a 48-channel semantic-context branch. Branch-specific transformations are then applied, followed by complementary spatial and channel modulation and final feature fusion. For DFAU, the input feature first predicts content-dependent offsets, then the offsets are fused with the base sampling grid, and finally bilinear grid sampling produces the aligned upsampled feature. For HSA, the normalized P5 sequence is compressed into N=64 hidden states. The gated hidden-state branch, hidden-state self-attention branch, and *D*-weighted state-residual branch are then fused. The fused hidden representation is projected, reconstructed using $C_{s}$, and reshaped to obtain the refined P5 feature.

### 3.8. Training Protocol and Evaluation Strategy

**Figure 5:**
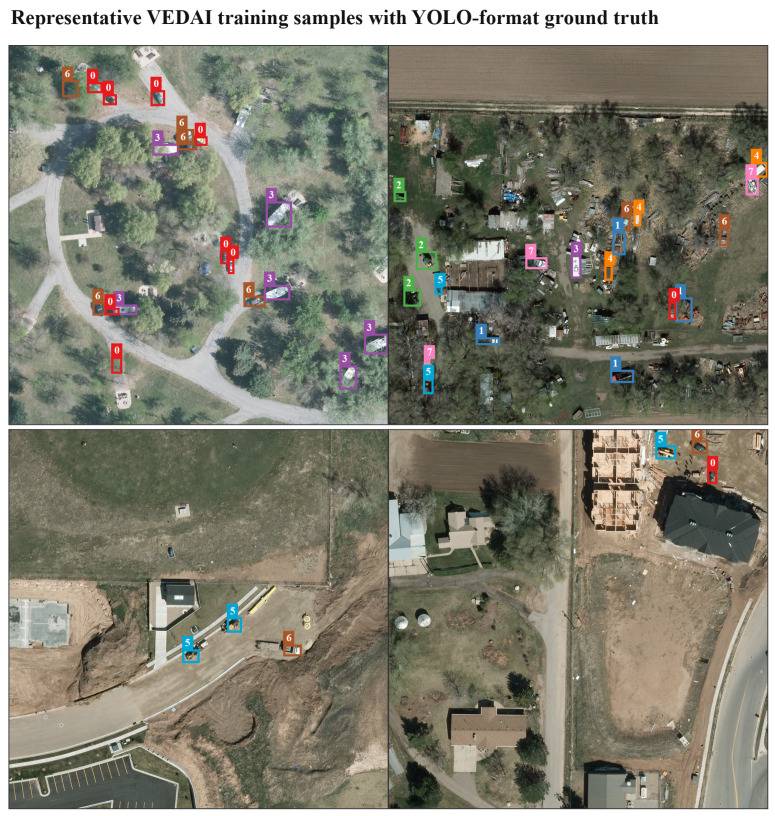
Representative VEDAI aerial-image samples with YOLO-format ground-truth annotations used in this study. Box colors distinguish vehicle classes, and labels 0–7 denote the YOLO class indices corresponding to id_1, id_2, id_4, id_5, id_9, id_10, id_11, and id_23, respectively. Overlapping labels reflect densely distributed ground-truth instances and do not affect the annotations.

All models are compared under the same data split, input size, and training epochs. The main experiments use the VEDAI eight-class aerial vehicle dataset, with an input size of 640×640 and 100 training epochs. DOTA-v1.0 HBB 1024-patch experiments are used for cross-dataset generalization validation to examine transfer behavior in broader aerial-image target detection scenarios. Representative training samples are shown in Figure 5.

For the VEDAI dataset, we used the eight-class vehicle detection setting, including id_1, id_2, id_4, id_5, id_9, id_10, id_11, and id_23. The dataset was converted into YOLO format and divided into 1000 training images and 250 validation images using a fixed split with seed 2. No separate independent test set was used in this study; therefore, all VEDAI results reported in this paper are obtained on the validation split. The same class setting and data split were used for YOLO11n, all ablation variants, and the final SFC-YOLO to ensure a controlled comparison.

To improve reproducibility, the main training hyperparameters used in the final reported experiments are summarized in Table 4. All final experiments reported in this manuscript used the SGD optimizer. The same basic augmentation settings were used across the checked paper-related runs.

The evaluation metrics include Precision, Recall, mAP50, parameter count, GFLOPs, and practical inference speed. Precision reflects false-alarm control, Recall reflects target retrieval ability, and mAP50 measures detection performance at an IoU threshold of 0.50. Parameter count represents model storage and parameter efficiency, GFLOPs reflect theoretical computation, and practical inference speed provides supplementary evidence for runtime behavior under a specific hardware and inference framework.

This work distinguishes repeated-run statistical comparison from auxiliary diagnostic ablation analysis. Five independent training runs are used for the YOLO11n baseline, the FCB-only configuration, the DFAU-only configuration, the FCB+DFAU configuration, and the final SFC-YOLO. All repeated runs use the same VEDAI data split, recorded seed setting, input size, optimizer, training schedule, augmentation configuration, and model definition, and each run is launched as an independent training process without resuming from another run. Mean and sample standard deviation are reported for Precision, Recall, and mAP50. The HSA-only, FCB+HSA, and DFAU+HSA variants are retained as auxiliary single-run diagnostic point estimates and are not used as formal statistical-significance evidence.

### 3.9. Methodological Summary

SFC-YOLO performs targeted enhancement for three key problems in aerial-image small vehicle detection: FCB compensates P5 high-level small-object features, DFAU improves P5-to-P4 cross-scale alignment, and HSA strengthens contextual interaction in the final P5 detection branch. The method is positioned as a parameter-efficient accuracy-enhanced framework rather than a FLOPs compression method. Therefore, the experiments jointly report detection accuracy, parameter count, theoretical computation, practical inference speed, and run-to-run variability for the final model and key ablation configurations.

## 4. Results

### 4.1. Main Comparison with YOLO11n

Table 5 presents the five-run mean results of YOLO11n and SFC-YOLO on the VEDAI eight-class vehicle detection task. Compared with YOLO11n, SFC-YOLO reduces the parameter count from 2.584 M to 2.466 M, corresponding to a 4.55% reduction. The mean Precision increases from 0.6022 to 0.6653, Recall increases from 0.5994 to 0.6079, and mAP50 increases from 0.6136 to 0.6523. These results indicate that the proposed structure improves average detection performance while reducing the parameter count.

It should be noted that the GFLOPs of SFC-YOLO increase from 6.3 to 8.4, corresponding to a 33.33% increase. Therefore, SFC-YOLO is not positioned as a theoretical FLOPs-compression model. Instead, it is an accuracy-enhanced model with better parameter efficiency, and its practical deployment should be evaluated together with measured inference speed.

### 4.2. Generalization Validation on DOTA-v1.0

To further examine cross-dataset generalization, DOTA-v1.0 was converted to horizontal bounding boxes and processed using a 1024×1024 sliding window with a stride of 824 pixels. This preprocessing produced 10,130 training patches and 3582 validation patches from the original 1411 training images and 458 validation images. This setting avoids severe small-object compression caused by directly resizing large DOTA images to 640×640. All DOTA runs use 100 epochs, batch size 4, and training input size 640. As shown in Table 6, the original large-image YOLO11n baseline obtains only 0.41636 mAP50, whereas the 1024-patch baseline reaches 0.69505 mAP50. Under the same DOTA HBB 1024-patch setting, SFC-YOLO improves mAP50 to 0.70033, indicating a modest cross-dataset gain under the patch protocol.

### 4.3. Repeated Experiments and Stability Analysis

To reduce the influence of isolated training outcomes, five independent runs were conducted for YOLO11n, FCB-only, DFAU-only, FCB+DFAU, and the complete SFC-YOLO. Table 7 reports the mean and sample standard deviation of Precision, Recall, and mAP50, together with the observed mAP50 range.

SFC-YOLO achieves an average mAP50 of 0.6523 with a standard deviation of 0.0117 and a range of 0.6409–0.6650. Its mAP50 run-to-run variability is lower than that of the FCB-only configuration, which obtains 0.6279 ± 0.0161, and the DFAU-only configuration, which obtains 0.6159 ± 0.0203. The variability of SFC-YOLO is approximately comparable to that of FCB+DFAU, whose mAP50 standard deviation is 0.0118. These results support the observation that the complete three-module configuration provides more consistent behavior than the independently evaluated single-module FCB and DFAU variants under the current protocol.

The observed variation is related to the difficulty of small-object detection on VEDAI and the numerical characteristics of GPU-based training. VEDAI contains many vehicles with limited pixel occupancy, while several categories exhibit sparse samples, weak textures, and similar appearances. Although all repeated runs used the same data split, recorded seed setting, input size, optimizer, augmentation configuration, and training schedule, complete bitwise determinism is not guaranteed because of nondeterministic GPU kernels and floating-point accumulation. For configurations containing DFAU, differentiable grid sampling may further amplify small numerical differences during optimization. In a relatively small and challenging aerial dataset, these minor differences can lead to measurable run-to-run variation. A separate systematic evaluation of longer training schedules or stronger regularization was not conducted in this study; these strategies will be investigated in future work. The reported statistics are descriptive repeated-run results; no formal claim of statistical significance is made.

### 4.4. Ablation Study

To analyze the independent contributions and combination effects of FCB, DFAU, and HSA, ablation experiments are conducted starting from YOLO11n. For the baseline, FCB-only, DFAU-only, FCB+DFAU, and complete SFC-YOLO configurations, Table 8 reports the five-run mean results. The HSA-only and the remaining two-module variants are retained as auxiliary single-run diagnostic point estimates.

The repeated-run results provide a different interpretation from the original isolated runs. The five-run mean mAP50 increases from 0.6136 for YOLO11n to 0.6279 with FCB and to 0.6159 with DFAU. Combining FCB and DFAU further increases the mean mAP50 to 0.6392. Compared with DFAU-only, FCB+DFAU improves the observed mean mAP50 by 0.0234, while compared with FCB-only, it improves mAP50 by 0.0114.

The original isolated DFAU result of approximately 0.6510 was close to the upper end of the five-run DFAU range of 0.6005–0.6511. In contrast, the original FCB+DFAU result of approximately 0.6280 was close to the lower end of its five-run range of 0.6275–0.6580. Therefore, the previously observed non-monotonic difference was mainly associated with run-to-run variation and does not provide evidence that FCB interferes with DFAU offset prediction. The repeated-run means instead indicate that feature compensation and dynamic alignment provide complementary improvements.

After HSA is introduced into the final P5 detection branch, the complete SFC-YOLO achieves a five-run mean mAP50 of 0.6523, which is 0.0131 higher than FCB+DFAU and 0.0386 higher than YOLO11n. Its mAP50 standard deviation is also lower than those of the independently evaluated FCB-only and DFAU-only variants and approximately equal to that of FCB+DFAU. These results support a progressive complementary trend among FCB, DFAU, and HSA, while the auxiliary single-run configurations are not used to claim formal statistical significance.

### 4.5. Per-Class Analysis

Table 9 reports per-class results for YOLO11n and SFC-YOLO in a representative single run, which is used for class-level diagnostic analysis rather than as the final averaged result. SFC-YOLO achieves clear gains on classes id_2, id_9, and id_10. Specifically, the AP50 of id_2 increases from 0.601 to 0.716, and the AP50 of id_9 increases from 0.359 to 0.577.

In contrast, the AP50 of id_23 decreases from 0.501 to 0.472. Its Precision increases from 0.595 to 0.682, whereas its Recall decreases from 0.436 to 0.333, indicating that SFC-YOLO applies more conservative filtering to this category: some false positives are suppressed, but additional true instances are missed.

To investigate this class-specific degradation, we further analyzed the VEDAI annotations. The id_23 class contains 132 training instances and 39 validation instances, for a total of 171 instances, ranking second among the eight classes in ascending instance count. This supports sample scarcity as a plausible contributor to the reduced Recall. However, its median bounding-box area is 1376.0 pixels^2^, ranking fifth among the eight classes from smallest to largest, and 27.5% of its instances have an area below 32×32 pixels. Therefore, the annotation statistics do not support unusually small object scale as the primary explanation for the degradation.

The available labels cannot directly quantify intra-class appearance variation or background ambiguity. These factors are therefore retained only as qualitative possibilities rather than established causes. Overall, the id_23 result is more plausibly related to limited training samples and conservative semantic filtering than to systematically smaller object size. Future work will investigate class-balanced sampling, category-specific augmentation, and loss reweighting to improve this difficult category.

### 4.6. Complexity and Practical Inference Speed Analysis

In addition to detection accuracy, model complexity and inference speed are important evaluation indicators for visual-sensor detection systems. SFC-YOLO reduces the parameter count from 2.584 M to 2.466 M but increases GFLOPs from 6.3 to 8.4. This result confirms that parameter efficiency, theoretical computation, and practical runtime efficiency are distinct properties. The additional computation is mainly introduced by the dynamic grid-sampling operation in DFAU and the hidden-state interaction in HSA.

The speed benchmark was conducted on an NVIDIA GeForce RTX 4070 Laptop GPU with 8188 MiB of memory, an Intel(R) Core(TM) i7-14700HX CPU, and 15.8 GB RAM. The software environment consisted of Microsoft Windows build 10.0.26200, Python 3.11.15, PyTorch 2.7.1+cu118, CUDA 11.8, cuDNN 9.1.0, Ultralytics 8.4.33, and NVIDIA driver 566.24. Neither TensorRT nor ONNX Runtime was used, and no model quantization, pruning, or other acceleration backend was applied.

YOLO11n and SFC-YOLO were evaluated under batch sizes 1 and 16 using the same VEDAI validation split and an input size of 640×640. For each model–batch condition, one complete warm-up pass was performed and excluded from the statistics, followed by five repeated validation passes. Table 10 reports the mean and sample standard deviation of the per-image inference latency, total latency, inference FPS, and total FPS.

Under batch size 1, the total latency increases from 8.4155±0.7557 ms for YOLO11n to 12.2608±0.1804 ms for SFC-YOLO, while total FPS decreases from 119.59±10.59 to 81.57±1.21. Under batch size 16, the total latency increases from 4.8445±0.2582 ms to 5.2822±0.1870 ms, and total FPS decreases from 206.93±11.85 to 189.50±6.57. Therefore, the repeated measurements do not support a runtime-throughput advantage for SFC-YOLO on the tested platform.

However, the relative total-latency increase decreases from approximately 45.7% at batch size 1 to approximately 9.0% at batch size 16. This narrower gap indicates that larger-batch GPU execution partially amortizes the overhead of dynamic sampling and hidden-state processing. Overall, SFC-YOLO should be interpreted as an accuracy-enhanced and parameter-reduced detector rather than a latency- or FLOP-optimized detector.

### 4.7. Visualization Analysis

**Figure 6:**
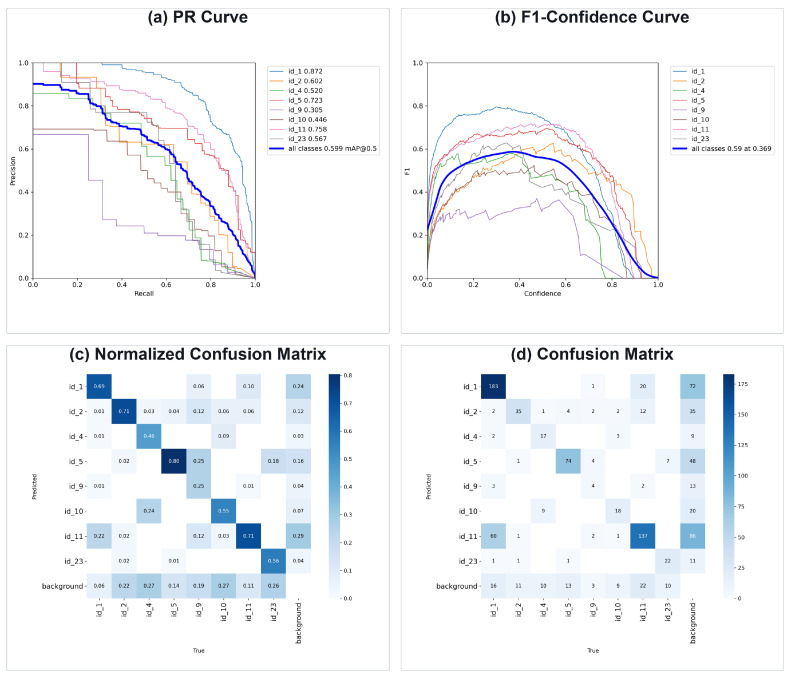
Quantitative diagnostic visualizations from the SFC-YOLO validation results: (**a**) PR curve; (**b**) F1-confidence curve; (**c**) normalized confusion matrix; and (**d**) confusion matrix.

**Figure 7:**
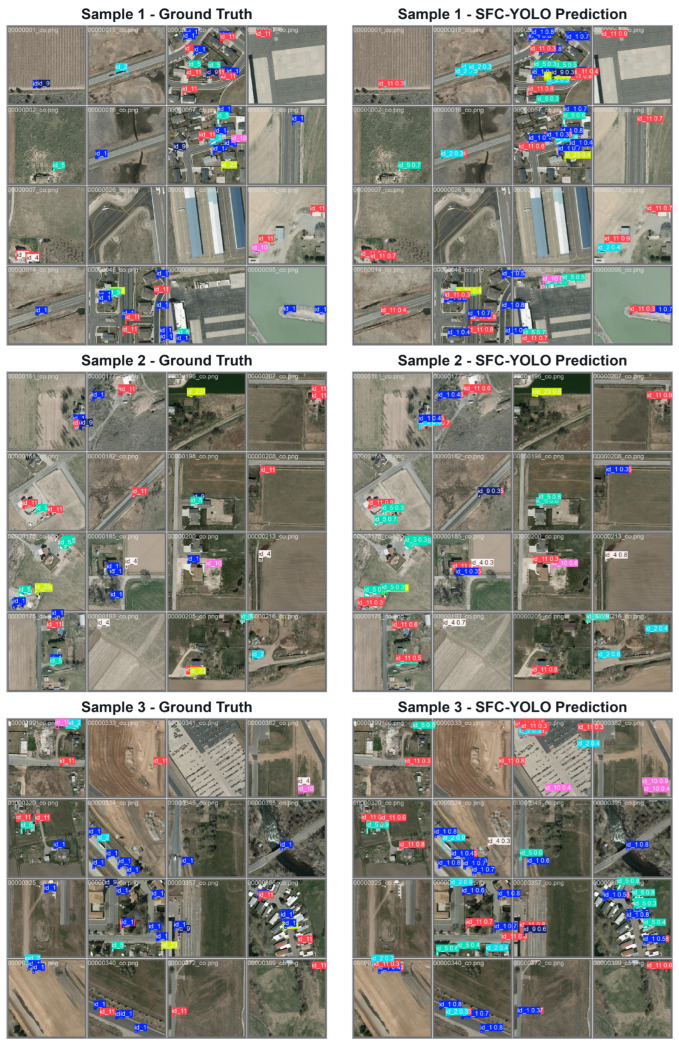
Qualitative validation examples arranged by sample, with ground-truth annotations in the left column and the corresponding SFC-YOLO predictions in the right column. Box colors distinguish vehicle classes; each prediction label contains the class identifier and confidence score. Label overlap in dense scenes reflects closely spaced detections and does not alter the underlying results.

**Figure 8:**
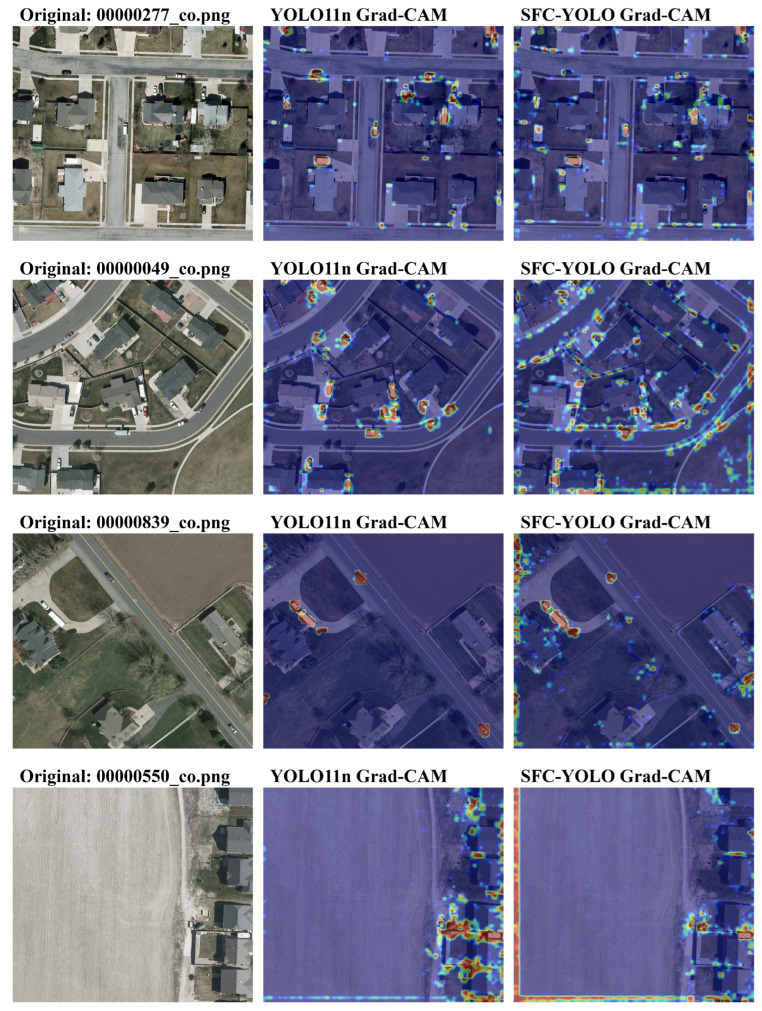
Grad-CAM heatmap comparison between YOLO11n and SFC-YOLO on representative VEDAI validation images. Blue regions indicate low activation, whereas green, yellow, and red regions indicate progressively stronger class-discriminative responses.

**Figure 9:**
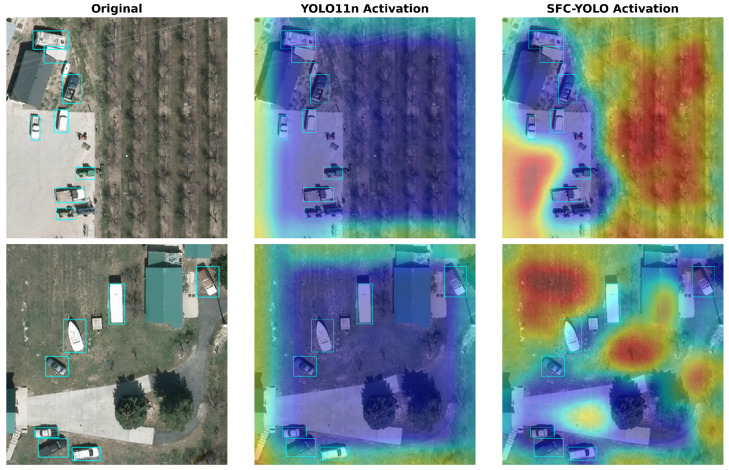
Intermediate activation comparison between YOLO11n and SFC-YOLO on representative VEDAI validation crops. The original crops are annotated with vehicle boxes to indicate small-object locations. Compared with the baseline, SFC-YOLO shows stronger activation responses in vehicle-containing local regions and nearby contextual structures, providing qualitative evidence of changed intermediate feature representations. Cyan boxes mark annotated vehicle locations, and blue-to-red colors indicate low-to-high activation intensity.

To assist interpretation of the experimental results, this work provides diagnostic visualizations from validation logs, as shown in Figure 6, Figure 7, Figure 8 and Figure 9. These visualizations include PR curves, F1-confidence curves, confusion matrices, validation-batch label and prediction visualizations, Grad-CAM heatmap comparisons, and intermediate activation comparisons between YOLO11n and SFC-YOLO. PR and F1-confidence curves show class-wise confidence behavior and overall diagnostic trends. Confusion matrices reveal inter-class misclassification patterns. Label and prediction visualizations show typical detection effects. Grad-CAM heatmaps are used to visualize class-discriminative regions and compare the spatial response differences between YOLO11n and SFC-YOLO on vehicle regions and complex background regions [52], supporting interpretation of how the proposed structures affect small-object feature responses.

To further complement the Grad-CAM analysis, we additionally compare intermediate activation responses between YOLO11n and SFC-YOLO on representative validation crops. As shown in Figure 9, the original crops are annotated with vehicle boxes to indicate the small-object locations. Compared with YOLO11n, SFC-YOLO shows stronger activation responses in vehicle-containing local regions and nearby contextual areas, suggesting that the proposed framework changes the intermediate feature representation for small-vehicle detection. Although this visualization does not isolate each module independently, it provides additional qualitative evidence for the overall feature-response difference between YOLO11n and SFC-YOLO.

## 5. Discussion

The experimental results show that SFC-YOLO achieves better average detection performance than YOLO11n on aerial vehicle detection. The improvement mainly comes from the three modules that target different bottlenecks. FCB is placed at the P5/32 high-level feature stage and strengthens small-vehicle representation in deep features through complementary modulation between a local-detail branch and a semantic-context branch. DFAU is placed in the P5-to-P4 upsampling path and improves alignment between high-level semantic responses and mid-level spatial structures through dynamic sampling. HSA is placed in the final P5 detection branch and enhances global high-level semantic interaction through hidden-state self-attention.

The repeated-run ablation results indicate that the modules provide complementary improvements for different error sources. The five-run mean mAP50 values are 0.6136 for YOLO11n, 0.6279 for FCB-only, 0.6159 for DFAU-only, 0.6392 for FCB+DFAU, and 0.6523 for the complete SFC-YOLO. Therefore, the original isolated difference between DFAU-only and FCB+DFAU was not sustained across repeated runs. The complete SFC-YOLO also shows lower mAP50 run-to-run variability than the independently evaluated FCB-only and DFAU-only variants, while its variability is approximately comparable to that of FCB+DFAU. These observations support the complementary roles of feature compensation, dynamic alignment, and hidden-state semantic interaction, without implying formal statistical significance.

From an efficiency perspective, SFC-YOLO reduces the parameter count but increases both GFLOPs and measured latency, confirming that parameter-count efficiency does not imply computational or runtime efficiency. In the five-run benchmark, SFC-YOLO is slower than YOLO11n under both batch sizes 1 and 16. Nevertheless, the relative total-latency gap decreases from approximately 45.7% at batch size 1 to 9.0% at batch size 16, indicating that larger-batch execution partially amortizes the overhead of DFAU and HSA on the tested GPU. Deployment decisions should therefore jointly consider parameter count, GFLOPs, latency, throughput, target hardware, and expected batch size.

A scope boundary of this study is that the quantitative comparison mainly focuses on YOLO11n and its controlled structural variants. This design was adopted to isolate the contribution of the proposed FCB, DFAU, and HSA modules under the same dataset split, input size, training schedule, and evaluation protocol. Although several lightweight or aerial-image detectors have been reported in the literature, direct numerical comparison across papers may be misleading because different studies often use different dataset splits, class definitions, image resolutions, training schedules, augmentation strategies, and evaluation settings. Therefore, broader comparison with recent lightweight aerial-image detectors under a unified training and evaluation pipeline is left as an important direction for future work.

There are also limitations. First, the gain on DOTA-v1.0 HBB 1024-patch validation is modest. This limited improvement may be related to the patch-based processing pipeline, the category characteristics of DOTA, and the transfer boundary of the proposed modules. The 1024-patch pipeline already reduces the scale gap between large aerial images and the network input size, which substantially improves the YOLO11n baseline and leaves less room for additional structural improvement. In addition, DOTA contains broader object categories and more diverse scene layouts than the vehicle-centered VEDAI setting, whereas SFC-YOLO is mainly designed for small vehicle detection. Moreover, the HBB conversion ignores object orientation information, which may limit the benefit of feature enhancement for rotated aerial objects. Therefore, the DOTA result should be interpreted as a preliminary cross-dataset validation under the HBB patch protocol rather than as strong evidence of broad generalization across all aerial-object categories.

Second, the current method focuses on horizontal bounding boxes and does not explicitly model object orientation. Since vehicles and other aerial objects often appear with arbitrary orientations and dense layouts in remote-sensing images, extending SFC-YOLO to oriented bounding boxes (OBBs) is an important future direction. A possible extension is to replace the current HBB detection head with a rotated-box prediction head, introduce angle-aware regression losses, and evaluate the method on oriented remote-sensing benchmarks.

Third, class-specific behavior remains a limitation. The annotation analysis shows that id_23 has the second-lowest instance count among the eight classes, whereas its median bounding-box area is not unusually small. This suggests that sample scarcity is a more plausible contributor to its reduced Recall than object scale. Future work will consider class-balanced sampling, category-specific augmentation, and loss reweighting to improve difficult or underrepresented categories.

Fourth, the current experiments are mainly based on standard aerial-image benchmarks and do not explicitly evaluate adverse weather, illumination changes, water splashes, hail, light snow, or other environmental disturbances. Such conditions may change vehicle appearance, background texture, and image-compression patterns, which can reduce the reliability of small-vehicle detection. Wiseman proposed a lightweight traffic-congestion monitoring method that analyzes JPEG/DCT block-level changes in the compression domain rather than relying only on decompressed RGB images [53]. The flooded-road splash and hail examples in Figure 5 and Figure 6 of that study show that adverse environmental conditions can change compression-domain responses and may lead to misclassification. This observation suggests that future work could investigate hybrid vision–compression frameworks, where SFC-YOLO provides deep visual localization and compression-domain cues provide complementary robustness indicators under complex aerial scenes.

Fifth, the current speed evaluation is conducted on a single workstation platform. Broader hardware-level benchmarking on embedded GPUs, edge devices, and different desktop GPUs will be needed to evaluate deployment robustness. Future work will therefore focus on broader cross-dataset validation, OBB-based detection, minority-class optimization, adverse-weather robustness, hybrid vision–compression modeling, and deployment evaluation on more hardware platforms.

## 6. Conclusions

This paper proposes SFC-YOLO, an accuracy-enhanced and parameter-efficient YOLO framework for small vehicle detection in aerial images. Based on YOLO11n, the method introduces FCB at the P5/32 high-level feature stage, DFAU in the P5-to-P4 upsampling path, and HSA in the final P5 detection branch. FCB improves small-object feature representation through complementary local-detail and semantic-context modulation; DFAU improves cross-scale feature alignment through dynamic offset sampling; and HSA strengthens high-level semantic interaction through hidden-state self-attention.

Experiments on the VEDAI eight-class dataset show that SFC-YOLO reduces the parameter count from 2.584 M to 2.466 M while achieving five-run mean Precision, Recall, and mAP50 values of 0.665, 0.608, and 0.652, respectively. Five repeated experiments yield an mAP50 of 0.6523 ± 0.0117, quantifying the run-to-run variation under the current training protocol. On the DOTA-v1.0 HBB 1024-patch validation, SFC-YOLO improves mAP50 from 0.69505 to 0.70033, suggesting that the method retains a performance gain under the patch-based cross-dataset setting. Future work will focus on broader cross-dataset evaluation, oriented bounding-box detection, and deployment optimization.

## Figures and Tables

**Table 1:** Key structural modifications in SFC-YOLO and their methodological motivations.

Module	Insertion Position	Primary Function	Bottleneck Addressed
FCB	P5/32 feature stage	High-level feature compensation	Weak detail after deep downsampling
DFAU	P5-to-P4 upsampling path	Content-adaptive feature alignment	Cross-scale spatial mismatch
HSA	Final P5 branch	Hidden-state semantic interaction	Limited context under complex backgrounds

**Table 2:** Architecture-level summary of the modified parts in SFC-YOLO.

Part	Network Position	Input and Output Dimensions	Parameter Count
Baseline YOLO11n	Three-scale detection framework	Input: 640×640 image; output: P3-, P4-, and P5-scale detections.	2.584 M total parameters.
FCB	P5/32 high-level feature stage	$R^{B\times C\times H_{5}\times W_{5}}\to R^{B\times C\times H_{5}\times W_{5}}$.	226,054 trainable parameters (0.226 M).
DFAU	First P5-to-P4 upsampling path	P5 feature at $H_{5}\times W_{5}$ → aligned feature at $2H_{5}\times 2W_{5}$.	8224 trainable parameters (0.008 M).
HSA	Final P5 detection branch	$R^{B\times C\times H_{5}\times W_{5}}\to R^{B\times C\times H_{5}\times W_{5}}$.	380,220 trainable parameters (0.380 M).
Final SFC-YOLO	YOLO11n with FCB, DFAU, and HSA	Input: 640×640 image; output: the original three-scale detection form.	2.466 M total parameters; 4.55% fewer than YOLO11n.

**Note:** The FCB, DFAU, and HSA values denote the trainable parameters contained in the corresponding module instances and are included in the final SFC-YOLO parameter budget.

**Table 3:** Implementation details of the proposed FCB, DFAU, and HSA modules.

Module	Insertion Position	Exact Implementation Configuration
FCB	P5/32 high-level feature stage	The C3k2-style wrapper receives and outputs 256-channel P5 features. Its internal C3k path contains two FCM cores. Each core processes a 64-channel hidden feature and divides it into a 16-channel local-detail branch and a 48-channel semantic-context branch. The local branch uses two 3×3 Conv–BN–SiLU layers followed by a 1×1 Conv–BN–SiLU projection; the semantic branch uses a 1×1 Conv–BN–SiLU projection. Spatial weighting uses 1×1 convolution, batch normalization, and Sigmoid. Channel weighting uses depthwise 3×3 convolution, global average pooling, and Sigmoid. The weighted branches are added and passed through a final 1×1 Conv–BN–SiLU projection.
DFAU	First P5-to-P4 upsampling path	The offset field is predicted by a 1×1 convolution and scaled by 0.25 before being added to the regular initial sampling pattern. The configuration is scale=2, style=lp, groups=4, and dyscope=False. Pixel-shuffled coordinates are used for grouped bilinear grid_sample with padding_mode=border and align_corners=False. The P4-to-P3 path retains nearest-neighbor upsampling.
HSA	Final P5 detection branch	The normalized P5 sequence is compressed into N=64 hidden states. The state-variable projection maps $d_{\mathrm{model}}$ to 3N channels. The hidden/gating projection maps $d_{\mathrm{model}}$ to $2d_{\mathrm{model}}$, and the QKV projection maps $d_{\mathrm{model}}$ to $3d_{\mathrm{model}}$. The attention-output projection preserves $d_{\mathrm{model}}$. The state-space expansion ratio is 1, four attention heads are used, both attention and projection dropout are 0, and the learnable attention coefficient λ is initialized to 0.

**Table 4:** Training hyperparameters used in the final reported experiments.

Setting	VEDAI Experiments	DOTA Original HBB	DOTA HBB-1024
Input size	640	640	640
Epochs	100	100	100
Batch size	16	4	4
Workers	4	2	1
Optimizer	SGD	SGD	SGD
Initial learning rate, lr0	0.01	0.01	0.01
Final learning-rate factor, lrf	0.01	0.01	0.01
Momentum	0.937	0.937	0.937
Weight decay	0.0005	0.0005	0.0005
Warm-up epochs	3.0	3.0	3.0
Mosaic	1.0	1.0	1.0
MixUp	0.0	0.0	0.0
Copy-paste	0.0	0.0	0.0
HSV-H/HSV-S/HSV-V	0.015/0.7/0.4	0.015/0.7/0.4	0.015/0.7/0.4
Scale/Translate/FlipLR	0.5/0.1/0.5	0.5/0.1/0.5	0.5/0.1/0.5
Recorded seed	2 for all VEDAI repeated runs	2	2

**Table 5:** Five-run mean comparison between YOLO11n and SFC-YOLO on the VEDAI eight-class dataset.

Model	Params/M	GFLOPs	Precision	Recall	mAP50
YOLO11n	2.584	6.3	0.6022	0.5994	0.6136
SFC-YOLO	2.466	8.4	0.6653	0.6079	0.6523
Gain	−4.55%	+33.33%	+0.0631	+0.0085	+0.0386

**Table 6:** Generalization validation on DOTA-v1.0 HBB with 1024×1024 sliding-window patches.

Setting	Model	Precision	Recall	mAP50
Original HBB	YOLO11n	0.72310	0.37849	0.41636
HBB-1024	YOLO11n	0.73867	0.67254	0.69505
HBB-1024	SFC-YOLO	0.73006	0.67542	0.70033
HBB-1024	Gain	−0.00861	+0.00288	+0.00528

**Table 7:** Five-run statistics for the baseline and key ablation configurations on VEDAI.

Model	Runs	Precision Mean ± Std	Recall Mean ± Std	mAP50 Mean ± Std	mAP50 Range
YOLO11n	5	0.6022 ± 0.0000	0.5994 ± 0.0000	0.6136 ± 0.0000	0.6136–0.6136
YOLO11n+FCB	5	0.6406 ± 0.0384	0.5946 ± 0.0370	0.6279 ± 0.0161	0.6085–0.6491
YOLO11n+DFAU	5	0.6183 ± 0.0597	0.5966 ± 0.0365	0.6159 ± 0.0203	0.6005–0.6511
YOLO11n+FCB+DFAU	5	0.6444 ± 0.0382	0.6099 ± 0.0235	0.6392 ± 0.0118	0.6275–0.6580
SFC-YOLO	5	0.6653 ± 0.0335	0.6079 ± 0.0282	0.6523 ± 0.0117	0.6409–0.6650

**Table 8:** Ablation study of FCB, DFAU, and HSA. Rows with five runs report mean values, whereas rows with one run are auxiliary diagnostic point estimates.

Model	Modules	Runs	Precision	Recall	mAP50
YOLO11n	None	5	0.6022	0.5994	0.6136
YOLO11n+FCB	FCB	5	0.6406	0.5946	0.6279
YOLO11n+DFAU	DFAU	5	0.6183	0.5966	0.6159
YOLO11n+HSA	HSA	1	0.6130	0.5940	0.6263
YOLO11n+FCB+DFAU	FCB+DFAU	5	0.6444	0.6099	0.6392
YOLO11n+FCB+HSA	FCB+HSA	1	0.6380	0.6080	0.6420
YOLO11n+DFAU+HSA	DFAU+HSA	1	0.6020	0.6240	0.6380
SFC-YOLO	FCB+DFAU+HSA	5	0.6653	0.6079	0.6523

**Table 9:** Per-class comparison between YOLO11n and SFC-YOLO in a representative single run. The “All” row reflects this representative run and is therefore different from the five-run average reported in Table 7.

Class	Model	Precision	Recall	AP50
id_1	YOLO11n	0.881	0.748	0.882
id_1	SFC-YOLO	0.896	0.739	0.885
id_2	YOLO11n	0.468	0.694	0.601
id_2	SFC-YOLO	0.549	0.714	0.716
id_4	YOLO11n	0.677	0.486	0.551
id_4	SFC-YOLO	0.773	0.459	0.584
id_5	YOLO11n	0.657	0.790	0.732
id_5	SFC-YOLO	0.789	0.650	0.747
id_9	YOLO11n	0.454	0.261	0.359
id_9	SFC-YOLO	0.721	0.485	0.577
id_10	YOLO11n	0.418	0.606	0.500
id_10	SFC-YOLO	0.467	0.485	0.556
id_11	YOLO11n	0.651	0.762	0.783
id_11	SFC-YOLO	0.719	0.725	0.783
id_23	YOLO11n	0.595	0.436	0.501
id_23	SFC-YOLO	0.682	0.333	0.472
All	YOLO11n	0.600	0.598	0.614
All	SFC-YOLO	0.699	0.574	0.665

**Table 10:** Five-run repeated inference-speed benchmark on the VEDAI validation split. Latency is reported in milliseconds per image, and all values are mean ± sample standard deviation.

Model	Batch	Inference Latency/ms	Total Latency/ms	Inference FPS	Total FPS
YOLO11n	1	6.5901±0.6017	8.4155±0.7557	152.75±13.83	119.59±10.59
SFC-YOLO	1	10.3868±0.1391	12.2608±0.1804	96.29±1.29	81.57±1.21
YOLO11n	16	3.2077±0.2440	4.8445±0.2582	313.36±26.45	206.93±11.85
SFC-YOLO	16	3.6785±0.2010	5.2822±0.1870	272.48±14.40	189.50±6.57

**Note:** Each condition was measured over five repeated validation passes after one excluded warm-up pass. Total latency is the sum of preprocessing, inference, and postprocessing latency in the default PyTorch/Ultralytics pipeline.

## Data Availability

The VEDAI and DOTA-v1.0 datasets used in this study are publicly available. The processed horizontal-bounding-box annotations, dataset split files, training configuration files, and experimental logs generated during this study are available from the corresponding author upon reasonable request.

## Abbreviations

The following abbreviations are used in this manuscript:

FCB	Feature Complementary Block
DFAU	Dynamic Feature Alignment Upsampling
HSA	Hidden-State Attention
HBB	Horizontal Bounding Box
OBB	Oriented Bounding Box
GFLOPs	Giga Floating-Point Operations
mAP	mean Average Precision
